# Green Packaging Films with Antioxidant Activity Based on Pectin and *Camellia sinensis* Leaf Extract

**DOI:** 10.3390/molecules29194699

**Published:** 2024-10-04

**Authors:** Renata Dobrucka, Mikołaj Pawlik, Marcin Szymański

**Affiliations:** 1 Department of Non-Food Products Quality and Packaging Development, Institute of Quality Science, Poznan University of Economics and Business, al. Niepodległości 10, 61-875 Poznan, Poland; mikoajpawlik5@gmail.com; 2 Center for Advanced Technologies, Adam Mickiewicz University in Poznan, Uniwersytetu Poznańskiego 10, 61-614 Poznan, Poland; marcin.szymanski@amu.edu.pl

**Keywords:** biodegradation, environmental safety, packaging films, pectin, green matcha

## Abstract

In the packaging materials sector, increasing globalization has created the need for increased efforts to develop consumer protection measures. Consequently, new packaging materials are being sought to replace petroleum-based materials in the future. For this reason, global awareness of the environmental problems associated with the use of synthetic and non-degradable packaging has increased the attention paid to bio-packaging based on natural and biodegradable polymers. The bio-packaging sector is developing innovations to address the sustainability issues facing the food packaging industry. Our research has shown that green matcha extract can be a promising source of antioxidants for the production of bioactive pectin films. This study further confirmed that green matcha extract can be a promising source of antioxidants for the production of bioactive pectin films. The antioxidant activity test showed high activity of films containing matcha extract. The antioxidant activity of films without matcha addition, P, PJ, PC, PJC, was negligible. The addition of matcha to the polymer matrix did not significantly affect the mechanical properties (TS, EB) of the films obtained. The addition of cellulose had the greatest effect on changing the mechanical properties. It caused a twofold increase in the mechanical properties of the obtained packaging films. The addition of matcha significantly improved the barrier properties (for PM films, the WVTR was 3.40 [g/m^2^d]; for PJM films the WVTR was 1.70 [g/m^2^d]). The green packaging films showed no toxic effects on the plants (*Phacelia tanacetifolia, Salvia hispanica, Brassica napus*) and invertebrates (*Daphnia pulex, Chaoborus, Chironomus aprilinus*) tested. The half-solubility time of the membranes in a solution mimicking gastric acid was also determined. The longest half-dissolution time of the films was about 2 min. Our research has therefore shown that the biodegradable and environmentally safe green packaging films with antioxidant activity that we have developed can be used as edible functional casings in the future, e.g., for sausages and other food products.

## 1. Introduction

Today, the packaging industry, and in particular the plastic packaging industry, faces a number of challenges as a result of the legislation contained in the directive on reducing the environmental impact of plastic packaging materials. Plastic food packaging accounts for more than 40% of all plastic waste worldwide, leading to a gradual accumulation of plastic in landfills and causing serious environmental concerns. It is estimated that the total amount of packaging waste generated will increase from 78 million metric tonnes in 2020 to 92 million metric tonnes in 2030 and 107 million tonnes in 2040 [1]. The excessive use of plastics and the improper disposal of non-biodegradable plastics cause severe environmental pollution, leading to concerns about the use of plastic packaging materials [2]. In addition, plastics are composed of a variety of reactive, toxic chemicals and can accumulate at higher trophic levels, causing human ingestion (in the form of microplastic contaminants) [3]. The degradation of petroleum-based plastics is very slow, starting with photodegradation and resulting in thermo-oxidative degradation. The energy of activation starts the fusion of oxygen atoms into polymers, which is provided by the ultra-violet rays of the sun. As a result, the plastic turns out to be brittle and breaks down into smaller pieces, which accumulate in plant and animal organisms.

The initiative put forward by the directive is also considered in the wider context of the European Union’s transition to a closed loop economy, which is one of the key areas of development for the European Union. In order to meet the expectations of the directive, the closed loop economy, and the zero waste approach, plastic packaging producers are facing huge changes, even a revolution of sorts. The search is therefore on for new packaging materials that will be able to replace petroleum-based materials in the future [4]. For this reason, the global awareness of the environmental problems associated with the use of synthetic and non-degradable packaging has increased the attention given to biopackaging based on natural and biodegradable polymers. To this end, many biopolymers can be good alternatives to conventional polymers due to their excellent gas barrier properties, mechanical properties and film-forming properties. Among the various known biopolymers, pectin can be a good choice due to its abundance and excellent physical and functional properties. Pectin’s gelation properties, as well as its biocompatibility and biodegradability, make pectin an ideal biomaterial for many applications [5]. Furthermore, pectin (labeled E440 in the EU) has been classified by the WHO/FAO Expert Committee as a functional food additive, without limitation of its intake level.

Pectins are a complex group of naturally occurring heteropolysaccharides in the cell walls and lamellae of all plants. Pectins can be extracted from a variety of natural sources. Most of these resources are fruit and vegetable peels, which can be considered a sustainable source for the extraction of this biopolymer, as they are usually discarded as waste during processing. Citrus peels and apple pomace are available in large quantities as by-products in food products. However, the growing demand and interest in exploiting the different functional properties of pectin allows the use of other by-products and wastes from food production and the agro-industry for pectin extraction. Despite the possibility of obtaining pectin from many plants on an industrial scale, pectin is mainly extracted from citrus and apple processing residues [6]. A second aspect of the use of these wastes is the high pectin content, which is 15–20% by weight in apple pomace dry matter and about 30% by weight in citrus dry matter [7]. It is also a waste management method that contributes to sustainability and environmental protection. Furthermore, pectin can be an excellent polymer matrix for edible films or coating materials for food packaging due to its biodegradability and environmentally friendliness. In addition, pectins have high water solubility, flexibility, and barrier properties, which are of great importance in the oxidation processes of stored food.

Several studies have been reported in the global literature on the preparation of pectin-based films for active packaging applications. Pectin was used to obtain films with red cabbage and beetroot extract [8]. Nisar et al. [9] recently developed a bioactive functional packaging film from citrus pectin with the addition of clove bud essential oil. The obtained film showed improved physical (water vapor barrier properties, hydrophobicity) and mechanical properties. Bernhardt et al. [10] obtained pectin films with the addition of maize husks. There were improvements in mechanical properties, water vapor barrier properties, hydrophobicity and antioxidant activity. On the other hand, Sood and Saini [11] reported the fabrication of a composite film based on pectin extracted from the peels of red pomelo. Casein and egg albumin were added as additional fillers. The resulting materials showed improved mechanical properties, water resistance and vapor barrier properties. The novelty in this paper is the inclusion of matcha green into the polymer matrix. It is novel because, as previously discussed, there is not much information available on the incorporation of matcha green tea into a polymer matrix to produce a packaging film with antioxidant properties and improved barrier properties.

**Figure 1:**
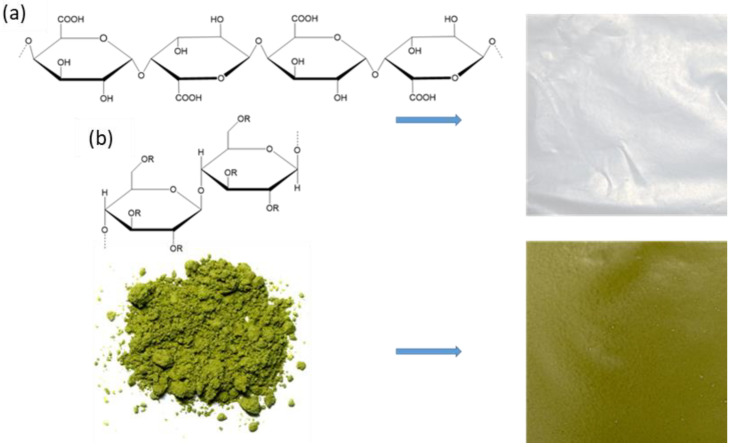
The scheme for the production of green packaging films based on pectin and *Camellia sinensis* leaf extract. (**a**) Pectin– (**b**) carboxymethylcellulose.

In the present work, films based on pectin and cellulose and matcha green tea *Camellia sinensis* leaf extract were prepared. Figure 1 shows a scheme for the preparation of these films. Matcha green tea (*Camellia sinensis* leaf extract) is a high-quality green tea that is a fine powder of tea leaves [12]. Matcha is grown in the dark (approximately 90%) by covering the growing fields with various materials. In this way, the cultivated tea leaves are harvested, washed, dried and then ground using stone mills to produce a powder known as matcha tea powder. For the consumption of matcha powder, the whole powder is mixed with hot water so that the entire contents of the leaves are consumed, not just the extract [13].

According to the literature, tea leaves are rich in various polyphenols, including catechins, tea flavins and flavonols. Matcha tea is a rich source of phenolic compounds, among which catechins account for 80% of all polyphenols. The main tea catechins are epicatechin (EC), epigallocatechin (EGC), epicatechin gallate (ECG) and epigallocatechin gallate (EGCG) [14]. Matcha has a relatively high caffeine content compared to other green teas. According to Koláčková et al. [15], the caffeine content of matcha ranges from 18.9 to 44.4 mg/g depending on the region of origin. In comparison, coffee beans contain 10.0–12.0 mg caffeine/g bean. Due to the presence of these active substances, matcha exhibits strong free radical scavenging activity, as evidenced by the increased free radical scavenging activity of 1,1-diphenyl-2-picrylhydrazyl (DPPH) and iron-ion-reducing antioxidant activity [16,17]. The biodegradable and environmental safety green packaging films with antioxidant activity based on pectin and *Camellia sinensis* leaf extract presented in this study are a response to the current search for materials that integrate into a circular economy.

## 2. Results and Discussion

### 2.1. GC-MS of Matcha Extract

In this study, we wanted to check the composition of matcha because, as reported by Devkota et al. [13], the chemical composition of matcha powder depends on various factors, such as growing conditions, processing, and consumption of the whole powder rather than the extract. Therefore, the composition of matcha was assessed using the ethanolic extract. Table 1 shows the results of the GC-MS study of the ethanolic extract of matcha. The GCMS study showed that the leading compound is caffeine (more than 80%), with benzoic acid (about 10%) 2,3-dimethyl-2,3-butanediol (3.28%), phytol acetate (2, 74%), (1aa,1bß,1cß,2aß,3aß,6aa,6ba,7a,8ß,8aa)-8-(acetylxy)-1,1a,1b,1c,2a,3,3a,6a,6b,7,8,8a-dodecahydro-3a,6b,8a-trihydroxy-2a-(hydroxymethyl)-1,1,5,7-tetramethyl-4H-cyclopropa[5′,6′]benz[1′,2′:7,8]azulene[5,6-b]oxiren-4-one (1.15%) and compounds present in trace amounts of less than 1%.

Matcha is rich in natural antioxidants such as polyphenols: various compounds that make up as much as 30% of the dry weight [18]. Polyphenols are powerful antioxidants, almost as effective as vitamins like vitamin C, vitamin E, carotene, and tocopherol. In matcha, catechins make up 90% of these polyphenols. The main four types of catechins are (-)-epicatechin (EC), (-)-epicatechin-3-gallate (ECG), (-)-epigallocatechin (EGC) and (-)-epigallocatechin-3-gallate (EGCG), where EGCG is the most abundant and active [19]. According to Jakubczyk et al. [16], matcha contains 44.8 mg/L of vitamin C, 1968.8 mg/L of flavonoids and 1765.1 mg/L of polyphenols in an infusion made with 1.75 g of plant material in 100 mL of water. In addition to these, there are many other biologically important molecules, such as flavonols (e.g., kemferol, quercetin, rutin), phenolic acids (protocatechuic acid, gallic acid, caffeic acid, synapinic acid, ellagic acid, etc.), amino acids (e.g., L-theanine, gamma aminobutyric acid (GABA)), methylxanthines (caffeine, theophylline, theobromine), chlorophyll and volatile compounds (e.g., pentanal, heptanal, 2-butanone) [13].

Due to their high number of phenolic hydroxyl groups, catechins exhibit excellent antioxidant activity by acting as a reducing agent, hydrogen/electron donor, single oxygen quencher and metal chelator. In consideration of the strong antioxidant activity of catechins, many researchers have used catechins in packaging matrices to produce antioxidant packaging films [20].

Due to the low probability of the MS spectra being consistent with the NIST database in the next studies, the authors purchased analytical standards to confirm the identification.

### 2.2. Antioxidant Activity of Green Packaging Films

As previously mentioned, matcha infusion has strong antioxidant properties thanks to the presence of m.in, catechins, and polyphenolic compounds. Our research showed that green matcha was characterized by values for DPPH IC50 = 0.052 mg/mL and FRAP IT0.5 = 0.33 mg/mL. We also evaluated the antioxidant properties of the obtained films. Table 2 and Table 3 show the results of antioxidant activity studies. Studies of antioxidant activity using both the DPPH radical method and the FRAP method showed high activity of the film, which included matcha extract. The antioxidant activity of the film without the addition of matcha, P, PJ, PC, PJC was negligible. Therefore, the addition of matcha significantly affected the antioxidant activity of the obtained films.

PCM and PJCM films with cellulose were characterized by lower antioxidant activity. Films without the addition of cellulose PM and PJM were characterized by higher antioxidant activity. Despite this, all films exhibited antioxidant activity. This is the answer to the search for potentially antioxidant materials for food packaging. Oxidation is one of the main factors causing spoilage and the deterioration of food quality. The presence of carboxyl groups in the conjugated ring structures of many compounds contained in plant derivatives allows the neutralization of free radicals or the decomposition of peroxides, leading to the inhibition of lipid peroxidation, which is one of the main causes of food degradation during processing and storage [21,22]. In recent years, antioxidant packaging has been identified as the most promising packaging technology for delaying the oxidation of food. Antioxidant packaging is usually made by adding synthetic materials to packaging dyes. Hence, natural additives with antioxidant potential are sought. The films we have developed have a high antioxidant potential and can therefore be used in food packaging [20].

The antioxidant activity of the standard substances (gallic and caffeic acid) determined by the DPPH radical method and the FRAP method was stronger than that of the *Camellia sinensis* leaf extract and the films obtained with this extract.

### 2.3. FTIR Analysis of Green Packaging Films

**Figure 2:**
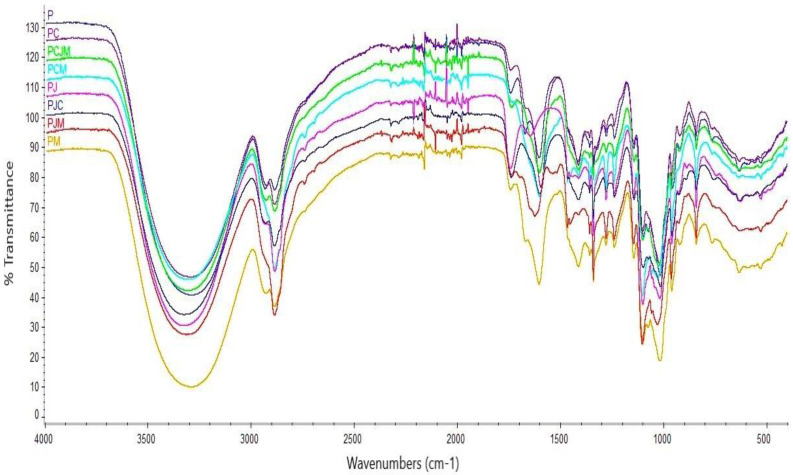
FTIR spectra of the films tested.

FT-IR analysis of the films was carried out to test chemical changes and functional group interactions between components in the films that would affect mechanical and other physical properties [23]. Figure 2 shows the FTIR spectra of the films studied. In all the spectra, intensive peaks at 3300 cm^−1^, 2900 cm^−1^, 2100 cm^−1^, 1600 cm^−1^, and 1000 cm^−1^ were observed. The observed wide range from 3300 cm^−1^ contains the fundamental stretching modes of hydroxyl groups (OH) due to water and carbohydrates. The intense peak at 2900 cm^−1^ was attributed to the stretching vibration of C–H. The strong peak at 1600 cm^−1^ is to be associated with the stretching of the carbonyl ester (C=O). In addition, a strong peak was observed to be absent at an absorption around 1749 cm^−1^, indicating a low-methoxy pectin [24]. A peak at 1000 cm^−1^ was also observed, which was attributed to C–O–C stretching of the saccharide structure of pectin [25]. The application of additives to the pectin matrix does not cause significant changes in the polymer structure, as demonstrated by FTIR spectra [26,27]. The difference in interactions between the film-forming substances will have an impact on the mechanical and barrier properties, which will be discussed below.

### 2.4. Mechanical and Barrier Properties of Green Packaging Films

Tensile strength indicates the maximum strength of the film, while elongation at break indicates the film’s prior stretch or elasticity. These properties are crucial for biodegradable films as they need to have sufficient strength. Moreover, mechanical properties are one of the most important properties of food packaging films. Because pectin is known to provide high mechanical and adhesive properties to plant cell walls, it is not surprising that it has considerable potential to be the main ingredient in a packaging film forming solution [11].

According to the literature [28], the mechanical properties of pectin films are largely influenced by the density of intermolecular networks (i.e., change in the distribution of pectins in the biopolymer network). Furthermore, lower tensile strength (TS) and higher elongation at break (EB) are usually associated with films composed of pure pectin. Our research showed similar results. For the film obtained from pure pectin, the TS results are 6.67 ± 1.31 [MPa] for citrus pectin (P), while for apple pectin (PJ), they are 7.00 ± 1.62 [MPa] (Table 4). We obtained a similar TS value for pectin films after adding matcha. The addition of matcha did not significantly affect the tensile strength. For the citrus pectin/matcha foil, the TS value was 7.00 ± 0.63 [MPa], and for the apple pectin/matcha foil, the TS value was 9.25 ± 0.20 [MPa]. However, the addition of cellulose significantly increased the TS value of the obtained films. Cellulose addition to prepared film samples had a significant effect (*p* < 0.05) on its mechanical properties. Thus, for the foil, citrus pectin/cellulose TS is 15.00 ± 3.69 [MPa], and for the foil, apple pectin/cellulose TS is 17.75 ± 2.01 [MPa]. This is due not only to the geometry but also to the formation of a rigid, continuous network of cellulose molecules linked by hydrogen bonds [29]. Moreover, the very similar chemical structure of cellulose and pectin leads to strong interactions between the functional groups of pectin molecules (including carboxyl and hydroxyl groups) and cellulose through interfacial hydrogen and ionic interactions [30]. It was very important to determine the optimal concentration of cellulose, because too high a concentration could reduce the interaction between cellulose and pectin and, as a result, cause its uneven dispersion [31].

In the process of preparing the film, a plasticizer (glycerine) was used, the presence of which resulted in a decrease in tensile strength and an increase in elongation at break (EB). Basically, this means that the film became more flexible and elastic and that the force required to break the film decreases [32]. For pure citrus (P) and apple (PJ) pectin films, the EB values are 25.99 ± 6.37 [%] and 30.96 ± 8.20 [%], respectively. The addition of cellulose to the polymer matrix resulted in a double increase in EB values for the films obtained. For the citrus pectin/cellulose films, the EB is 56.42 ± 37.56 [%], while for the apple pectin/cellulose films the EB is 60.59 ± 1.94 [%], respectively. In addition to the addition of a plasticizer causing an increase in the flexibility of the film. This effect is due to the good interaction between pectin and cellulose, which results in an efficient transfer of the applied stress and, consequently, a homogeneous distribution of the introduced stress and a reduction in stress concentration [33]. The addition of matcha to the pectin/cellulose polymer matrix did not result in significant changes in the elongation at break. However, pectin/matcha/cellulose films were characterized by a twofold increase in EB values. Thus, for the citrus pectin/cellulose/matcha films, the EB value was 52.97 ± 11.60 [%]. For the apple pectin/cellulose/matcha film, on the other hand, the EB was 58.47 ± 3.51 [%]. Based on the results, it was observed that the addition of matcha to the polymer matrix did not significantly affect the mechanical properties of the films obtained. The addition of cellulose to the pectin matrix had the greatest effect on the tensile strength (TS) and elongation at break (EB) changes. This resulted in a twice as much increase in the mechanical properties of the obtained packaging films.

**Figure 3:**
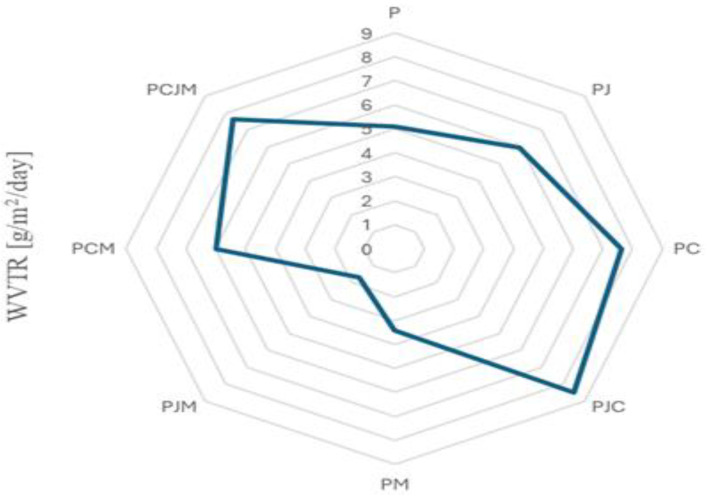
WVTR diagram for green packaging films.

The shelf life of food is directly connected to the permeation of water vapor between the food and the outside environment. This is a very important function of food packaging to minimize vapor transmission as rapidly as possible [5]. Therefore, it was very important in our study to evaluate the manufactured film in terms of vapor transmission. Pure citrus and apple pectin film had a vapor transmission rate value of 5.10 ± 0.01 [g/m^2^d] and 5.94 ± 0.00 [g/m^2^d], respectively. This indicates similar water vapor permeability to commercial, petroleum-based packaging materials such as HDPE. The barrier properties were significantly improved by the addition of matcha, and so for the citrus pectin/matcha film, the WVTR value was 3.40 ± 0.01 [g/m^2^d], and for the apple pectin/matcha film, it was 1.70 ± 0.00 [g/m^2^d]. This indicates a significant improvement in the barrier properties of the apple pectin film. This shows the very good high barrier properties of these films. This can be attributed to the presence of polyphenolic compounds present in the matcha, which result in improved barrier by building a dense cross-linking network of the polymer matrix. Figure 3 shows the WVTR diagram for green packaging films. Thus, the introduction of matcha into the polymer matrix improves the water barrier properties and increases the antioxidant potential, which is a crucial aspect for packaging films [34].

The application of cellulose did not improve the barrier properties of pectin films, even though the presence of cellulose nanocrystals is thought to increase the curvature of pectin films, resulting in slower water vapor diffusion processes and therefore lower permeability [35]. Indeed, for apple pectin/cellulose films, the WVTR value was 8.49 ± 0.00 [g/m^2^d], while for citrus pectin/cellulose films, it was 7.64 ± 0.02 [g/m^2^d].

### 2.5. Biotoxicity Results of Green Packaging Films

The use of higher plants is considered the most reliable and cost-effective test material for phytotoxicity testing, which has been endorsed by numerous international environmental regulatory organizations such as the United Nations Environment Program (UNEP), the World Health Organization (WHO) and the United States Environmental Protection Agency (USEPA) [36]. In the present study, phyto-toxicity/stimulation testing was conducted on films dissolved in water. The concentration of the stock solutions of all film samples was equal and was 20 mg/mL. The optimal concentration was determined experimentally by a series of analytical tests. We used plant seeds for the study: *Phacelia tanacetifolia, Salvia hispanica* and *Brassica napus.* Few studies of this type exist in the literature. Table 5, Table 6 and Table 7 show the mean value of shoot growth inhibitory activity for the film solution (inhibitory [values at +] or stimulatory [values at −] shoot growth), standard deviation and coefficient of variation for the measurement using rape, phacelia and chia, respectively.

The PCJM film solution (54.2%), PJ (53.1%) and PM (50.9%) proved to be the most inhibitory to oilseed rape growth, while the PC film solution (18.3%) and P (20.3%) were the least inhibitory (more than twice as weak). The high inhibitory activity values are most likely dictated by the sensitivity of oilseed rape to the various components present in the substrate.

Compared to phacelia, the PJ solution (30.8%) and PCM solution (27.0%) were the most inhibitory to shoot growth, while the PC film solution (13.6%) was the least inhibitory. The inhibitory activity values in each case were lower than for oilseed rape, with only the P film solution acting at a similar level (20.3% for oilseed rape and 19.2% for phacelia).

The results of inhibitory activity against chia appeared to be the most variable. The film solutions PCM, PJ, P, and PJM showed an inhibitory effect on shoot growth, while the film PC, PCJ, PCJM, and PM had a stimulatory effect on plant growth.

**Figure 4:**
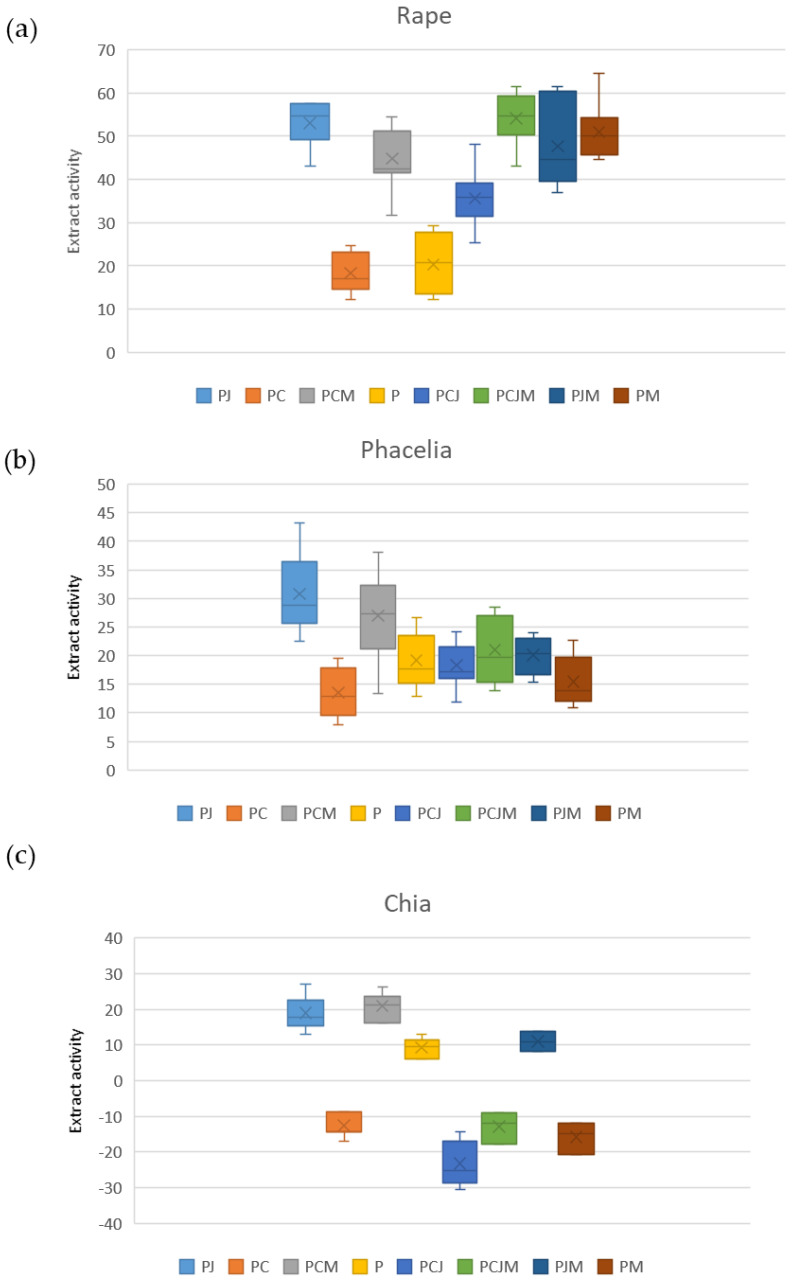
Graph of average foil activity values in relation to: (**a**) rape; (**b**) phacelia; (**c**) chia.

Figure 4 graphically shows the distribution of the mean shoot growth inhibitory activities of the liquid films with standard deviations for rape, phacelia and chia.

The influence of the film solution on the test plants rape, phacelia, and chia proved to be very different, caused by the different sensitivity of the plant to the components present. The greatest differences in the response of the test plants to the films tested occurred in the case of chia, depending on whether the film tested there was an inhibition or stimulation of shoot growth. The most stable reply was obtained for phacelia. Its inhibition activity ranged from about 13 to about 30.

### 2.6. Biotoxicity Tests on Invertebrates

**Figure 5:**
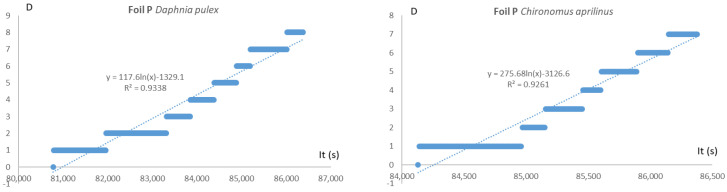

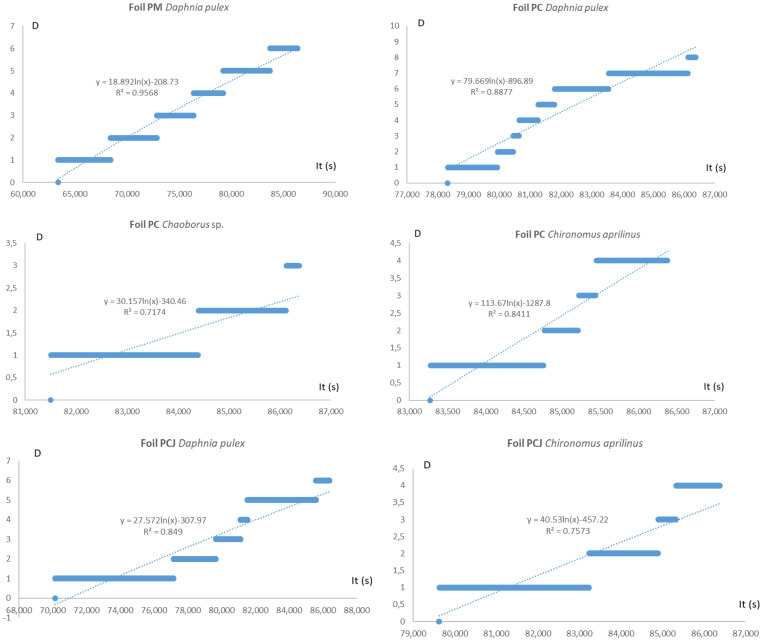
Graphs of the number of dead test organisms versus time, logarithmic function equations and the correlation coefficient R^2^.

Table 8 shows the time after which half-death of the test organisms was determined on the basis of the logarithmic function of the number of dead organisms against time, presented in seconds. Figure 5 shows graphs of the correlation of the number of dead test organisms with time, the equation of the logarithmic function, and the correlation coefficient R^2^.

The toxic effects of the film solution on the test invertebrate organisms proved to be low. Daphnia were found to be the most sensitive to film solutions (films P, PC, PJC–IT50 less than 24 h) and the least sensitive to worms, with only the PC sample showing a weak toxic effect (IT50 greater than 24 h).

### 2.7. Biodegradation Study of Green Packaging Films

Currently, intensive research is being conducted on the development of new materials that could replace plastics, which are produced on the basis of biodegradable polymers. Basically, the mechanism of polymer biodegradation, understood as a process of gradual decomposition, varies and depends on the type of polymer. The process as a whole involves reducing the chain, eliminating fragments, and lowering the molecular weight. The result is the formation of simple chemical compounds [37]. Traditional synthetic plastics are very resistant to degradation, and the lifespan of most plastics is estimated to be hundreds or even thousands of years, depending on the properties of the plastic and the surrounding environmental conditions [38]. The solution may be biopolymers based on which packaging materials are extruded.

In our research, we decided to investigate the biodegradation of developed materials using. This research is motivated by the fact that the problem of the biodegradation of biopolymer-based materials is also not fully recognized. This is an extremely important subject, as the biodegradability of new packaging materials is the basis for environmental protection, for example, in terms of the elimination of large volumes of post-consumer waste and the use of the natural resources of the Earth. Even though the biological decomposition of industrial materials of plant origin is a common phenomenon, the biodegradation process itself is very complex and dependent on many factors. In the case of multicomponent materials, the biodegradation process may or may not be the final stage of biological decomposition [38]. We conducted the study using demineralized water and an aqueous hydrochloric acid solution simulating gastric acid juice. Our films can provide edible functional films, e.g., for sausages or other food products.

**Figure 6:**
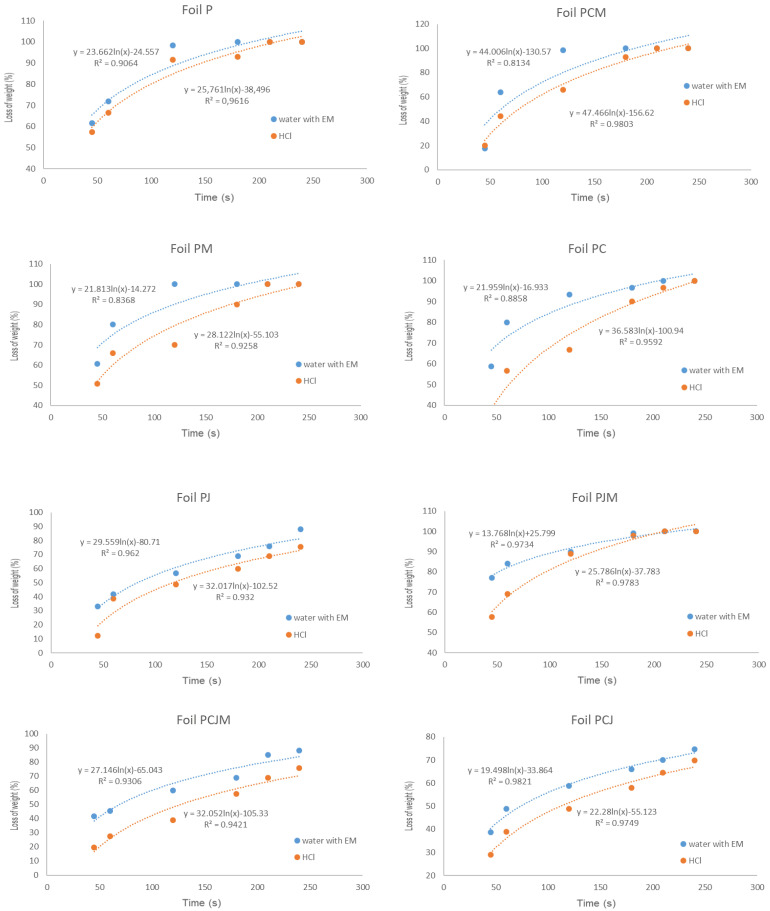
Graphs of the time dependence of the weight loss during solution in water with EM preparation and hydrochloric acid solution at pH = 2.

Figure 6 presents graphs of the dependence of weight loss on dissolution in water with EM and in hydrochloric acid solution at pH = 2, while Table 9 shows the solubility coefficient values of the tested films.

The determination of the half-solubility time of the film in hydrochloric acid solution at pH = 2 (mimicking gastric acid) made it possible to determine how long a given film would dissolve in the stomach. The hydrochloric acid environment resulted in a longer dissolution time for the film compared to the solubility in the presence of the EM preparation. The films P, PM, and PJM would dissolve the fastest in the stomach, while PCJM, PJ and PJC would take the longest. However, the longest half-life of the films dissolved in acid; i.e., about 2 min is small enough not to pose digestion problems. The preparation EM EmFarma Plus is a composition of live, beneficial non-GMO micro-organisms produced by natural fermentation processes. This preparation increases the decomposition of organic matter and improves the composting process. The dissolution process of the film in the EM preparation solution (10 mL of the preparation and 1000 mL of demineralized water) was much faster than in the hydrochloric acid solution. There was up to a fivefold reduction in dissolution time for PJM film and almost a threefold reduction for PC film. The film solubility was least affected by the EM formulation on PCM (−1.28-fold), P (−1.35-fold) and PJ (−1.41-fold) films.

### 2.8. SEM Analysis of Green Packaging Films

**Figure 7:**
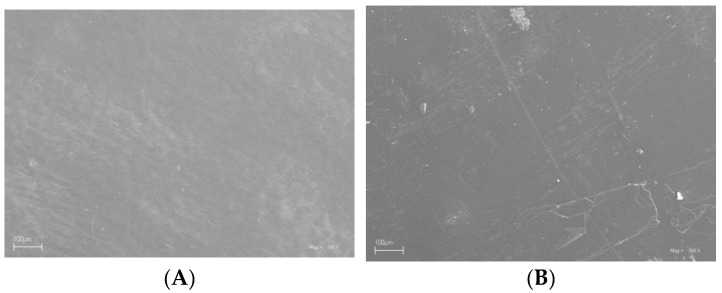

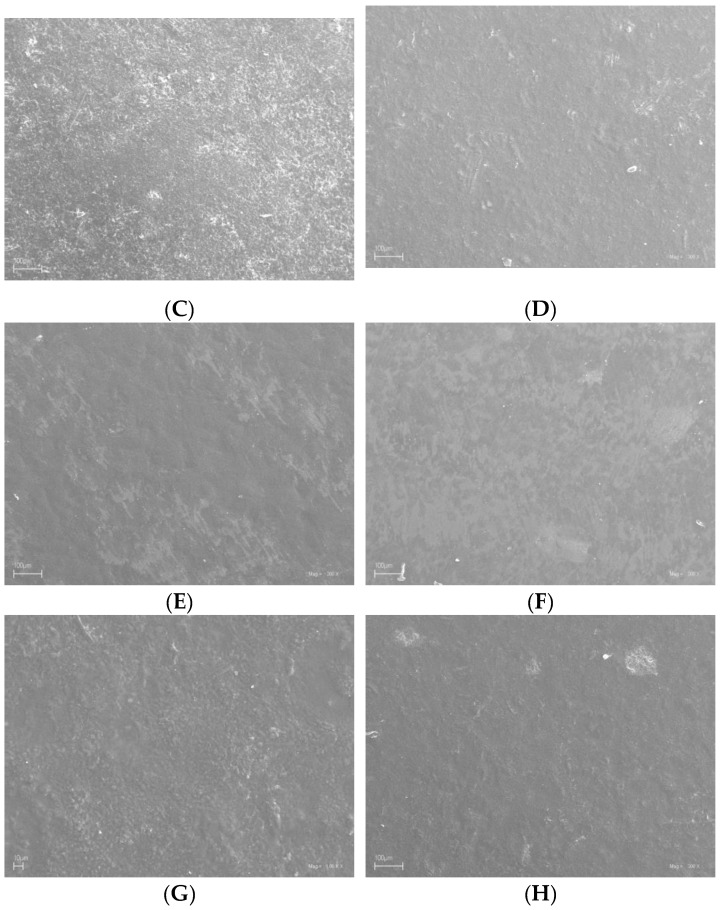
SEM images of prepared green packaging films: (**A**) P, (**B**) PC, (**C**) PM, (**D**) PCM, (**E**) PJ, (**F**) PCJ, (**G**) PJM, and (**H**) PCJM.

Scanning electron microscopy was used to examine the surface of the produced films. Figure 7 shows photos for (A) P, (B) PC, (C) PM, (D) PCM, (E) PJ, (F) PCJ, (G) PJM, and (H) PCJM foils taken at a scale of 100 um. For all foils, regardless of whether they contained matcha, the surface was smooth and not rough. Moreover, no cracks or phase separation was found on the film surfaces, suggesting a coherent matrix. Furthermore, our samples showed a continuous microstructure. In summary, the structure of a film is closely related to the interaction between its component materials. This in turn has far-reaching consequences for its various properties.

## 3. Materials and Methods

### 3.1. Materials

All chemical reagents (ethanol gradient grade; Water suitable for LC/MS, LiChrosolv; DPPH (1,1-Diphenyl-2-picrylhydrazyl, Free Radical) and TPTZ (2,4,6-tris(2-pyridyl)-1,3,5-triazines)) were purchased from Merck. The matcha used was produced by (BIO Matcha Tea Deli FOOD, manufacturer: Radziów Sp.z o.o.). Apple and citrus pectin (from Batom). Phytotoxicity tests were conducted using demineralized water (from J.T. Baker, Phillipsburg, NJ, USA) and Aerosil 200 (Evonik Resource Efficiency GmbH, Essen, Germany; CAS No. 112945-52-5). Seeds used in the study were *Phacelia tanacetifolia* Benth. Stala PL210/03/56/435A (Granum Sp.j.), *Salvia hispanica*–Chia (GutBio), and *Brassica napus* variety Berny (producer: Agrosimex, Błędów, Poland). For biotoxicity tests on invertebrates, the following were used (Producer: IT-Ichthyo Trophic sp. z o.o.): *Daphnia pulex*; *Chaoborus* sp. (larva); and *Chironomus aprilinus* (larva). For film degradation studies, the preparation of effective microorganisms EM EmFarma Plus (ProBiotics Polska Sp.z o.o.) was used.

### 3.2. Preparation of Green Packaging Films

Matcha extract was prepared from 1 g matcha with 100 ml distilled water. The resulting mixture was heated at 90 °C for about 1 h, stirring continuously, over heat. The extract was then mixed with a 1% pectin solution containing glycerol (0.5% *w*/*w*) at a ratio of 1:1. The obtained solutions were put under magnetic stirring at 500 rpm for 30 min at 90 °C. Also, solutions of 1% carboxymethylcellulose and pectin containing glycerol (0.5% *w*/*w*) were prepared and combined at a ratio of 1:1. Matcha extract was added to the prepared solution at a ratio of 1:1. The obtained solutions were put under magnetic stirring at 500 rpm for 30 min at 90 °C. Each film-forming solution was dried at 25 °C for 48 h in order to produce a thin film. The samples were produced without the addition of extract: P (citrus pectin), PJ (apple pectin), PC (citrus pectin/carboxymethylcellulose), and PJC (apple pectin/carboxymethylcellulose). Film samples with matcha were obtained: PM (citrus pectin/ matcha), PJM (apple pectin/ matcha),PCM (citrus pectin/ carboxymethylcellulose/ matcha) and PCJM (apple pectin/ carboxymethylcellulose/ matcha).

### 3.3. GC-MS Analysis

To 1.0 g of matcha tea, 4 mL of ethanol was added, heated for 5 min at boiling point on a heating bowl, then cooled and filtered through a 0.20 µm syringe filter. The filtrate was used for the GC-MS study. The study was performed on a Bruker gas chromatograph with mass detection, with the following parameters: electron energy 70 eV; ion source at 200 °C; silica column—VF-5 ms (30 m × 0.25 mm × 0.39); df = 0.25 μm; carrier gas—helium; gas flow rates ^−1^ mL/min. Temperature program: enable coolant at 50.0 °C, coolant timeout 20.00 min, stabilization time 0.50 min; temperature 60.0 °C, hold 3.00 min, total 3.00 min; temperature 280.0 °C, rate 10.0 °C min^−1^, hold 35.00 min, total 60.00 min. The identification of compounds was based on comparison of their retention times as well as mass spectra with standards from NIST.

### 3.4. Antioxidant Activity

An aqueous film solution of 20 mg/mL was used to test antioxidant activity. In the determination, dilutions of the stock solution suitable for the measurement method were prepared.

### 3.5. Antioxidant Activity with DPPH

The determination of antioxidant activity was performed spectrophotometrically against a solution of the DPPH radical. The measurement was performed at λ_max_ = 515 nm in 1 cm thick plastic cuvettes. The free radical reduction capacity of DPPH was determined using the following formula:

$$A_{a}=\left(A_{0}-\frac{A_{i}}{A_{0}}\right)\times 100\%$$

where *A_a_*—antioxidant activity [%]; *A_i_*—mean absorbance of the test solution *A*_0_—mean absorbance of DPPH solution. A total of 0.2 mL of the test sample and 1.4 mL of the DPPH solution were each added to 2 mL test tubes (Eppendorf type), the solution was mixed and allowed to stand in a dark place for 30 min, then the absorbance of the DPPH solution used was measured (5 replicates). The 0.2 mL solution of demineralized water with 1.4 mL of ethanol was used as a reference sample. The DPPH solution was prepared one hour before the tests. A weighed amount of the reagent was dissolved in ethanol and then transferred to a volumetric flask wrapped in aluminum foil. The concentration was selected empirically, assuming that the absorbance of the DPPH solution was about 1.0 (approximately 0.006 g DPPH per 100mL ethanol). The tests were conducted in an air-conditioned room at a temperature of 22 °C.

### 3.6. Antioxidant Activity by FRAP

Determination was carried out by spectrophotometry using the reagent TPTZ (2,4,6-tris(2-pyridyl)-1,3,5-triazines). Absorbance was measured at λ = 593 nm. Five replicates were made for each concentration prepared. A total of 0.1 mL of the solution to be analyzed and 1.5 mL of the FRAP mixture were each added to 2 mL tubes (Eppendorf type). The FRAP mixture was obtained by mixing 25 mL of acetate buffer at pH = 3.6, 2.5 mL of TPTZ solution in 40 mM HCl and 2.5 mL of 20 mM FeCl_3_·6H_2_O aqueous solution. The whole was then incubated on a shaker for 30 min at 37 °C. After this time, the absorbance at λ = 593 nm was measured. The equation of the curve (A = aC + b) was determined, and from this the concentration was calculated for an absorbance of A = 0.500. The tests were conducted in an air-conditioned room at a temperature of 22 °C.

### 3.7. FTIR

FTIR analysis was carried out using a Thermo Scientific Nicolet iS50 FTIR spectrometer (Thermo Fisher Scientific, Waltham, MA, USA)**.**

### 3.8. Mechanical Properties and Characterizantion of Green Packaging Films

Film mechanical properties were measured using an Instron universal testing machine (model 5965, Instron, Norwood, MA, USA) according to ASTMD882-12. Samples were cut into 1.5 cm × 10 cm strips and conditioned at 25 °C and 50% relative humidity for at least 48 h. The test speed was 100 mm/min. The average measurements from ten samples for mechanical properties, including tensile strength and elongation at break, are reported. The water vapor transmission rate (WVTR) for the green packaging material was determined in accordance with ISO 2528:2017 [38]. Containers were measured containing 10 g of anhydrous calcium chloride were covered with a film sample, sealed and placed in a desiccator containing saturated sodium chloride solution (RH = 65%, T = 23 °C).

### 3.9. Biotoxicity Study Using Plants

A 2.00 g sample of Aerosil 200 was weighed in a 250 mL beaker, 2 mL of demineralized film was dissolved in water (concentration 20 mg/mL), 20 mL of demineralized water was added, and it was mixed until homogeneous. The obtained Aerosil suspension was then transferred into 12 cm square Petri dishes. Selected seeds of *Phacelia tanacetifolia*, *Salvia hispanica*, *Brassica napus* were evenly applied to the leveled surface of the pad. The prepared samples were incubated at 23 °C for 120 h in a dark place. The plants were then gently removed from the dish and shoot growth was measured. Aerosil 200 with demineralized water (2 g/22 mL water) was used as a reference sample. On the basis of the measured values of shoot growth in relation to the control trial, the shoot growth inhibition activity of the test film solution (A) was calculated (100 × (length of growth of individual seed–average length of control trial)/average length of control trial). The negative values of the solution activity indicated growth stimulation, while positive values indicated a shoot growth inhibition effect.

### 3.10. Biotoxicity Study Using Selected Invertebrates

To petri dishes of 4 cm diameter with 10 daphnia, chironomid larvae or anglerfish larvae in 3 mL of aquarium water, 1 mL of dissolved film (20 mg/mL) was added. The slides were placed on a multimedia projector equipped with a camera. A 24 h image fixation cycle was set every 10 s. At the end of the study, the collected multimedia material was analyzed.

### 3.11. Biodegradation Study

Samples of 10 × 35 mm were prepared from a sheet of modified film and placed in 250 mL beakers. The following were added in parallel: 150 mL of an EM preparation solution prepared according to the manufacturer’s instructions and an aqueous hydrochloric acid solution of pH = 2 to mimic gastric juice. Incubation was carried out at 23 °C for 45, 60, 120, 180, 210, and 240 s, after which the film residue was air-dried and weighed. The weight difference before and after degradation and the percentage weight loss were calculated.

### 3.12. SEM Analysis

Microscopic research was carried out using an Evo 40 scanning electron microscope (Zeiss, Oberkochen, Germany) operating in beam mode at 20 kV with a secondary electron detector.

### 3.13. Statistical Analysis

The results of this study were presented as mean ± standard deviation, and each measurement was taken in a minimum of three replicates. Statistical evaluations were performed by one-way analysis of variance (ANOVA) using Statistica 13. The statistical significance was defined at the 95% confidence level. The method of least squares was used to calculate the antioxidant activity from DPPH and FRAP. The directional coefficient of the straight line a and the coefficient of deviation b were determined for the equation of the calibration curve y = a × x + b, followed by the rectilinear correlation coefficient R^2^, and the standard deviation of the straight line from the formula:

$$SD=\frac{\sqrt{\left(1-R^{2}\right)\times \sum y^{2}}}{n-1}$$

where R^2^—linear correlation coefficient; *y*—parameter value *y*; (*n* − 1)—number of degrees of freedom.

In order to determine the accuracy of the measurements, the coefficient of variation was calculated from the formula:

$$CV=\frac{SD}{\bar{y}}\times 100\%$$

where *ȳ*—average value of parameter *y*; *SD*—standard deviation.

The analysis of the results of the shoot growth inhibitory activity measurements for the phytotoxicity test consisted of calculating the mean value, standard deviation and coefficient of variation. EXCEL 2019 and STATISTICA12 were used for statistical calculations.

## 4. Conclusions

Due to the observed global progress, and consequently increased food production, the development of international transport, new packaging technologies have been sought for years to better protect food quality. Packaging made of materials with antioxidant activity containing natural antioxidants is one such solution. Compared to synthetic antioxidants, natural antioxidants such as herbs and spices are of great interest because of their safety and health characteristics. Moreover, recent strategies have focused on the development of biodegradable materials for use as environmentally friendly food packaging and coating. Natural plant antioxidants can be considered a safer and cost-effective alternative to synthetic antioxidants. In our study, we used green matcha, which is rich in active substances that show antioxidant activity. The study confirmed that green matcha extract can be a promising antioxidant source for the production of bioactive pectin films. A study of the antioxidant activity showed the high activity of the films containing matcha extract. The antioxidant activity of films without matcha added, P, PJ, PC, PJC, was negligible. In the case of strength tests, we observed that the addition of matcha did not significantly affect the tensile strength. The greatest effect on the change in tensile strength (TS) and elongation at break (EB) was due to the addition of matcha. It resulted in a two-fold increase in the mechanical properties of the resulting packaging films. In the case of the evaluation of the barrier properties of the films against water vapor, we observed that the addition of matcha significantly improved the properties of the pectin/matcha films. For the citrus pectin/matcha film, the WVTR value was 3.40 ± 0.01 [g/m^2^d], and for the apple pectin/matcha film it was 1.70 ± 0.00 [g/m^2^d]. It was extremely important to assess the environmental safety of the films. The developed films showed no toxicity to plants: Phacelia tanacetifolia, Salvia hispanica and Brassica napus. No toxic effects of the films were observed on the test invertebrate organisms: *Daphnia pulex*, *Chaoborus* sp., *Chironomus aprilinus*. This study is particularly important in the development of innovative packaging materials, which are a large group of materials. As mentioned earlier, the management of packaging waste is very important. Also very important is their impact on the natural environment, i.e., the so-called environmentally safe management of packaging waste. This knowledge is important at the design stage of such packaging or even material. This also applies to the now important recycled packaging. This is because it is well known that recycled products may contain more harmful substances than non-recycled products, potentially determining risks to human health and ecosystems when migrating into environmental elements. Moreover, our films can act as edible functional films for e.g., sausages, fruit or other food products, where they can be part of a product with health-promoting values. This is an alternative to the petroleum-based plastic casings currently in use, which are not easy for humans to digest and, in addition, may suffer from migration due to the presence of various additives in their manufacture. The solutions we propose are a natural and ecological alternative to the materials currently used for packaging food products.

## Figures and Tables

**Table 1:** GC-MS test results of ethanol matcha extract.

Peak RT (min)	Area	Quantity (%)	Compound Name	Formula	NIST Probability
4.523	5.27 × 10^7^	3.28	2,3-dimethyl-2,3-butanediol	C6H14O2	75.3
10.505	1.66 × 10^8^	10.33	benzoic acid	C7H6O2	44.4
18.158	4.40 ×10^7^	2.74	phytol, acetate	C22H42O2	14.8
18.213	8.62 × 10^6^	0.54	3,7,11-trimethyl-1-dodecanol	C15H32O	24.8
18.669	1.29 × 10^9^	80.43	caffeine	C8H10N4O2	98.1
22.339	6.32 × 10^6^	0.39	2-tridecanone	C13H26O	5.0
23.059	2.21 × 10^6^	0.14	9,10-secocholesta-5,7,10(19)-triene-3,24,25-triol, (3ß,5Z,7E)-	C27H44O3	13.7
24.071	1.85 × 10^7^	1.15	4H-cyclopropa[5′,6′]benz[1′,2′:7,8]azuleno[5,6-b]oxiren-4-one, 8-(acetyloxy)-1,1a,1b,1c,2a,3,3a,6a,6b,7,8,8a-dodecahydro-3a,6b,8a-trihydroxy-2a-(hydroxymethyl)-1,1,5,7-tetramethyl-, (1aa,1bß,1cß,2aß,3aß,6aa,6ba,7a,8ß,8aa)-	C22H30O8	37.1
26.712	1.63 × 10^7^	1.01	2,6,10,14,18-pentamethyl-2,6,10,14,18-eicosapentaene	C25H42	8.0

**Table 2:** Results of antioxidant activity tests of the aqueous solution of the tested films determined by the DPPH radical method.

Sample	IC50 (mg/mL)	y = a × x + b		R^2^	S **	CV *** (%)
		a	b			
P	472.72 *	0.1045	0.6195	0.8050	0.1661	10.6
PJ	55.40 *	0.8898	0.7074	0.9934	0.1866	2.1
PC	1808.55 *	0.0276	0.1687	0.8819	0.0341	8.2
PJC	89.87 *	0.5586	−0.1952	0.8953	0.4410	9.1
PM	0.48	3.7982	31.3983	0.9381	2.7134	5.5
PJM	0.68	64.7152	5.6797	0.8662	3.3348	9.4
PCM	0.64	48.5432	18.7414	0.9384	2.3592	5.7
PCJM	0.77	50.5942	10.8127	0.8582	3.1256	9.1
P_PR	0.052	897.3275	2.8946	0.8172	4.1169	11.3
gallic acid	0.032	1410.25	5.051	0.9832	1.0105	2.6
caffeic acid	0.024	2133.81	−1.840	0.9862	1.5879	2.5

* Theoretical value calculated from extrapolation. ** Standard deviation of a line. *** Coefficient of variation.

**Table 3:** Results of antioxidant activity tests of the aqueous solution of the tested films determined by the FRAP method.

Sample	FRAP Methods					
	IC0.5 (mg/mL)	a	b	R^2^	S	CV (%)
P	not demonstrated					
PJ	not demonstrated					
PC	not demonstrated					
PJC	not demonstrated					
PM	0.76	0.5054	0.1165	0.9994	0.0029	0.6
PJM	0.94	0.3859	0.1366	0.9994	0.0030	0.6
PCM	0.83	0.4450	0.1320	0.9980	0.0058	1.1
PCJM	0.97	0.3635	0.1467	0.9975	0.0073	1.4
P_PR	0.33	1.2296	0.1001	0.9982	0.0038	1.1
gallic acid	0.043	8.8324	0.1174	0.9951	0.0097	1.7
caffeic acid	0.036	9.0122	0.1777	0.9986	0.0052	0.9

**Table 4:** Mechanical properties of green films.

Sample	TS [MPa]	EB [%]
P	6.67 ^a^ ±1.31	25.99 ^a^ ± 6.37
PJ	7.00 ^a^ ±1.62	30.96 ^b^ ± 8.20
PC	15.00 ^b^ ± 3.69	56.42 ^c^ ± 37.56
PJC	17.75 ^b^ ± 2.01	60.59 ^c^ ± 1.94
PM	7.00 ^a^ ± 0.63	32.82 ^b^ ± 7.21
PJM	9.25 ^a^ ± 0.20	27.48 ^a^ ± 1.02
PCM	17.17 ^b^ ± 2.71	52.97 ^c^ ± 11.60
PCJM	15.13 ^b^ ± 1.94	58.47 ^c^ ± 3.51

Different letters indicate statistically significant differences (*p* < 0.05) in the properties of the prepared packaging materials. The values are represented as a mean ± standard deviation.

**Table 5:** Average value of activity inhibiting shoot growth for the foil solution, standard deviation and coefficient of variation for rapeseed.

Rape	PJ	PC	PCM	P	PCJ	PCJM	PJM	PM
Average activity (%)	53.1	18.3	44.8	20.3	35.7	54.2	47.7	50.9
SD *	5.1	4.8	6.8	6.6	6.4	5.7	9.6	6.1
CV **	9.7	26.1	15.2	32.5	18.0	10.5	20.2	11.9

* Standard deviation. ** Coefficient of variation.

**Table 6:** Average value of activity inhibiting shoot growth for the foil solution, standard deviation and coefficient of variation for phacelia.

Phacelia	PJ	PC	PCM	P	PCJ	PCJM	PJM	PM
Average activity (%)	30.8	13.6	27.0	19.2	18.4	21.0	20.1	15.4
SD	7.0	3.9	7.1	5.1	3.8	5.8	3.1	4.2
CV	22.7	28.6	26.4	26.4	20.9	27.5	15.3	27.3

**Table 7:** Average value of the shoot growth inhibitory activity for the foil solution, standard deviation and coefficient of variation for chia.

Chia	PJ	PC	PCM	P	PCJ	PCJM	PJM	PM
Average activity (%)	18.9	−12.7	21.0	9.3	−23.3	−12.9	11.0	−15.8
SD	4.2	2.9	3.6	2.8	5.9	3.8	2.3	3.6
CV	22.1	−23.1	17.0	29.7	−25.3	−29.8	21.4	−22.8

**Table 8:** Dead time of half of the test organisms tested.

Sample	T (sek)		
	*Daphnia pulex*	*Chaoborus* sp. (larwa)	*Chironomus aprilinus*
P	84,490	n.tox.	85,781
PJ	n.tox.	n.tox	n.tox
PC	82,493	94,408 *	86,966 *
PJC	85,051	n.tox.	89,715 *
PM	81,900	n.tox.	n.tox.
PJM	n.tox.	n.tox.	n.tox.
PCM	n.tox.	n.tox.	n.tox.
PCJM	n.tox.	n.tox.	n.tox.
P_PR	n.tox.	n.tox.	n.tox.

* Extrapolation (death of organisms over 24 h calculated from the curve).

**Table 9:** The IT50 half-solubility values of the tested films.

Sample	IT50 for Water with Prep. EM	IT50 for Hydrochloric Acid Solution	Reduced Dissolution Time
P	23	31	−1.35
PJ	83	117	−1.41
PC	21	62	−2.95
PJC	74	112	−1.51
PM	19	42	−2.21
PJM	6	30	−5.00
PCM	61	78	−1.28
PCJM	69	127	−1.84

## Data Availability

The original contributions presented in the study are included in the article, further inquiries can be directed to the corresponding author.

## References

[1] Diop C.I.K., Beltran S., Sanz M.T., Garcia-Tojal J., Trigo-Lopez M. (2023). Designing bilayered composite films by direct agar/chitosan and citric acid-crosslinked PVA/agar layer-by-layer casting for packaging applications. Food Hydrocoll..

[2] Zhang W., Azizi-Lalabadi M., Jafarzadeh S., Jafari S.M. (2023). Starch-gelatin blend films: A promising approach for high-performance degradable food packaging. Carbohydr. Polym..

[3] Butler I.P., Banta R.A., Tyuftin A.A., Holmes J., Pathania S., Kerry J. (2023). Pectin as a biopolymer source for packaging films using a circular economy approach: Origins, extraction, structure and films properties. Food Packag. Shelf Life.

[4] Alessandroni L., Caprioli G., Faiella F., Fiorini D., Galli R., Huang X., Sagratini G. (2022). A shelf-life study for the evaluation of a new biopackaging to preserve the quality of organic chicken meat. Food Chem..

[5] Karim R., Nahar K., Zohora F.T., Islam M.M., Bhuiyan R.H., Jahan M.S., Shaikh M.A. (2022). Pectin from lemon and mango peel: Extraction, characterisation and application in biodegradable film. Carbohydr. Polym. Technol. Appl..

[6] Das I., Arora A. (2021). Kinetics and mechanistic models of solid-liquid extraction of pectin using advance green techniques—A review. Food Hydrocoll..

[7] Adetunji L.R., Adekunle A., Orsat V., Raghavan V. (2017). Advances in the pectin production process using novel extraction techniques: A review. Food Hydrocoll..

[8] Otálora González C.M., De’Nobili M.D., Rojas A.M., Basanta M.F., Gerschenson L.N. (2021). Development of functional pectin edible films with fillers obtained from red cabbage and beetroot. Int. J. Food Sci. Technol..

[9] Nisar T., Wang Z.C., Yang X., Tian Y., Iqbal M., Guo Y. (2018). Characterization of citrus pectin films integrated with clove bud essential oil: Physical, thermal, barrier, antioxidant and antibacterial properties. Int. J. Biol. Macromol..

[10] Bernhardt D.C., Pérez C.D., Fissore E.N., De’Nobili M.D., Rojas A.M. (2017). Pectin-based composite film: Effect of corn husk fiber concentration on their properties. Carbohydr. Polym..

[11] Sood A., Saini C.S. (2022). Red pomelo peel pectin based edible composite films: Effect of pectin incorporation on mechanical, structural, morphological and thermal properties of composite films. Food Hydrocoll..

[12] Unno K., Furushima D., Hamamoto S., Iguchi K., Yamada H., Morita A., Nakamura Y. (2019). Stress-reducing effect of cookies containing matcha green tea: Essential ratio among theanine, arginine, caffeine and epigallocatechin gallate. Heliyon.

[13] Devkota H.P., Gaire B.P., Hori K., Subedi L., Adhikari-Devkota A., Belwal T., Kurauchi Y. (2021). The science of matcha: Bioactive compounds, analytical techniques and biological properties. Trends Food Sci. Technol..

[14] Sugimoto K., Matsuoka Y., Sakai K., Fujiya N., Fujii H., Mano J.I. (2021). Catechins in green tea powder (matcha) are heat-stable scavengers of acrolein, a lipid peroxide-derived reactive carbonyl species. Food Chem..

[15] Koláčková T., Kolofiková K., Sytařová I., Snopek L., Sumczynski D., Orsavová J. (2020). Matcha tea: Analysis of nutritional composition, phenolics and antioxidant activity. Plant Foods Hum. Nutr..

[16] Jakubczyk K., Dec K., Kałduńska J., Kawczuga D., Kochman J., Janda K. (2020). Reactive oxygen species-sources, functions, oxidative damage. Pol. Merkur. Lek. Organ Pol. Tow. Lek..

[17] Basak S., Mukherjee I., Das T.K. (2024). Injectable biocompatible RAFT mediated nitroxide nanogels: A robust ROS-reduction antioxidant approach. Colloids Surf. B Biointerfaces.

[18] Kurleto K., Kurowski G., Laskowska B., Malinowska M., Sikora E., Vogt O. (2013). Wpływ warunków parzenia na zawartość antyoksydantów w naparach różnych rodzajów herbat. Wiadomości Chem..

[19] Sokary S., Al-Asmakh M., Zakaria Z., Bawadi H. (2023). The therapeutic potential of matcha tea: A critical review on human and animal studies. Curr. Res. Food Sci..

[20] Yong H., Wang Z., Huang J., Liu J. (2024). Preparation, characterization and application of antioxidant packaging films based on chitosan-epicatechin gallate conjugates with different substitution degrees. Int. J. Biol. Macromol..

[21] Barzan G., Sacco A., Giovannozzi A.M., Portesi C., Schiavone C., Salafranca J., Rossi A.M. (2024). Development of innovative antioxidant food packaging systems based on natural extracts from food industry waste and Moringa oleifera leaves. Food Chem..

[22] Jiang B., Yue H., Fu X., Wang J., Feng Y., Li D., Liu C., Feng Z. (2024). One-step high efficiency separation of prolyl endopeptidase from Aspergillus niger and its application. Int. J. Biol. Macromol..

[23] Zeng F., Ye Y., Liu J., Fei P. (2023). Intelligent pH indicator composite film based on pectin/chitosan incorporated with black rice anthocyanins for meat freshness monitoring. Food Chem. X.

[24] Yong H., Liu J. (2020). Recent advances in the preparation, physical and functional properties, and applications of anthocyanins-based active and intelligent packaging films. Food Packag. Shelf Life.

[25] Jahromi M., Niakousari M., Golmakani M.T., Mohammadifar M.A. (2020). Physicochemical and structural characterization of sodium caseinate based film-forming solutions and edible films as affected by high methoxyl pectin. Int. J. Biol. Macromol..

[26] Nastasi J.R., Fitzgerald M.A., Kontogiorgos V. (2023). Tuning the mechanical properties of pectin films with polyphenol-rich plant extracts. Int. J. Biol. Macromol..

[27] Samir A.M.A.S., Alloin F., Sanchez J.Y., Dufresne A. (2004). Cross-linked nanocomposite polymer electrolytes reinforced with cellulose whiskers. Macromolecules.

[28] Cao X., Chen Y., Chang P.R., Stumborg M., Huneault M.A. (2008). Green composites reinforced with hemp nanocrystals in plasticized starch. J. Appl. Polym. Sci..

[29] Chaichi M., Hashemi M., Badii F., Mohammadi A. (2017). Preparation and characterization of a novel bionanocomposite edible film based on pectin and crystalline nanocellulose. Carbohydr. Polym..

[30] Galus S., Uchanski P., Lenart A. (2013). Colour, mechanical properties and water vapour permeability of pectin films. Acta Agrophysica.

[31] Kanagaraj S., Varanda F.R., Zhil’tsova T.V., Oliveira M.S., Simões J.A. (2007). Mechanical properties of high density polyethylene/carbon nanotube composites. Compos. Sci. Technol..

[32] Kurabetta L.K., Masti S.P., Eelager M.P., Gunaki M.N., Madihalli S., Hunashyal A.A., Kadapure A.J. (2023). Physicochemical and antioxidant properties of tannic acid crosslinked cationic starch/chitosan based active films for ladyfinger packaging application. Int. J. Biol. Macromol..

[33] Sanchez-Garcia M.D., Gimenez E., Lagaron J.M. (2008). Morphology and barrier properties of solvent cast composites of thermoplastic biopolymers and purified cellulose fibers. Carbohydr. Polym..

[34] Kumar V., Shahi S.K., Ferreira L.F.R., Bilal M., Biswas J.K., Bulgariu L. (2021). Detection and characterization of refractory organic and inorganic pollutants discharged in biomethanated distillery effluent and their phytotoxicity, cytotoxicity, and genotoxicity assessment using *Phaseolus aureus* L. and *Allium cepa* L. Environ. Res..

[35] Szymanski M., Nowicka J., Dobrucka R. (2024). Biodegradability and biotoxicity of the modified starch matrix with biologically synthesized ZnO nanoparticles. Environ. Prog. Sustain. Energy.

[36] Zhang K., Hamidian A.H., Tubić A., Zhang Y., Fang J.K., Wu C., Lam P.K. (2021). Understanding plastic degradation and microplastic formation in the environment: A review. Environ. Pollut..

[37] Czaja-Jagielska N., Domka F. (2007). Badania nad wykorzystaniem bakterii glebowych Bacillus licheniformis oraz bakterii osadu czynnego w procesie biodegradacji celulozy i folii zawierających celulozę. Polimery.

[38] (2017). Determination of Water Vapour Transmission Rate (WVTR)—Gravimetric (Dish) Method.

